# Motivators and deterrents in choosing a career in psychiatry; making the most of psychiatry school events

**DOI:** 10.1192/bjo.2021.405

**Published:** 2021-06-18

**Authors:** Nosheen Kazmi, Emily Lewis, Catarina Cardoso Rodrigues dos Santos, Sahana Olety

**Affiliations:** 1 ST4 CAMHS, Pennine Care NHS Foundation Trust; 2 Locum ST, Cheshire and Wirral Partnership NHS Foundation Trust; 3 ST4 Forensic Psychiatrist, Greater Manchester Mental Health; 4 Consultant Child and Adolescent Psychiatrist, UK, Pennine Care NHS Foundation Trust

## Abstract

**Aims:**

In response to the Royal College of Psychiatrists’ recruitment strategy, a bi-annual Psychiatry School event was set up in the North West of England. The Psychiatry School aims to inspire medical students and foundation doctors to choose a career in Psychiatry with two days of workshops on different sub-specialties and various aspects of the career pathway. A previous service evaluation has shown attending the event improves attitudes towards psychiatry.

The aim is to assess whether improving attitudes to psychiatry has been sustained and gain a clearer understanding of the motivators and deterrents in choosing a career in Psychiatry to better inform future events.

**Method:**

An online questionnaire about positive and negative aspects of psychiatry was sent to attendees of the Autumn North West Psychiatry School 2020 before and after the event.

**Result:**

The total number of completed questionnaires was 62.

53.6% people were considering applying for core psychiatry training prior to the event and this rose to 85.3% in the post event questionnaire.

Motivators for a career in psychiatry prior to the event included having a better holistic understanding of patients and wide range of sub-specialities. There was a common theme of interest in research opportunities. Dynamic patient-doctor relationship, exploring issues in depth and treating diverse populations were key motivators.

It is encouraging to note that 100% responders felt their positive views on psychiatry were validated.

The majority of deterrents were disregarded and attendees felt positive about choosing a career in psychiatry.

**Conclusion:**

Following the event, the only negative view on a career in Psychiatry was the concern about the potential impact on one's own mental health. This is an important issue (highlighted in the Royal College of Psychiatrists Position Statement) that deserves consideration at future events to highlight potential effects on Psychiatrists wellbeing and how these can be avoided or mitigated.

The wide variety of sub-specialities and opportunities for research were key areas that motivated attendees and we will continue to deliver engaging workshops around these themes.

